# Supramolecular Precursor Strategy to Construct g-C_3_N_4_/Silica Hybrid Nanosheets for Photocatalytic Degradation of Dye and Antibiotic Pollutants

**DOI:** 10.3390/nano12183108

**Published:** 2022-09-07

**Authors:** Yongsheng Yu, Jinghan Wang, Zhaoli Yan, Qiangshan Jing, Peng Liu, Bing Xu

**Affiliations:** 1 Henan Province Key Laboratory of Utilization of Non-Metallic Mineral in the South of Henan, College of Chemistry and Chemical Engineering, Xinyang Normal University, Xinyang 464000, China; 2 School of Chemistry and Chemical Engineering, Henan Polytechnic University, Jiaozuo 454000, China

**Keywords:** g-C_3_N_4_, supramolecular precursor, silica, hybrid nanosheets, photocatalysis

## Abstract

To construct a highly active g-C_3_N_4_ (CN)/silica hybrid nanosystem, the supramolecular precursor strategy of introducing melamine–cyanuric acid (MCA) by synergistically using micromolecular melamine (m) and urea (u) for CN nanostructure construction on the silica nanosheets (SiNSs) surface was researched. The results showed that the introduction of MCA supramolecular aggregates promoted the generation of ordered CN nanostructures attached to SiNSs, and the morphology of the CN nanostructure could be regulated through the m/u mass ratio. When the ratio is equal to 1/30, a typical g-C_3_N_4_/silica hybrid nanosheet (mu-CN/SiNSs-3) was successfully prepared, which showed the ultra-high photocatalytic activity for Rhodamine B dye degradation within 25 min with an apparent rate constant of 0.186 min^−1^, owing to the large surface area of highly dispersed and ordered CN nanosheets, a strong interaction between CN and SiNSs, high photogenerated carriers separation efficiency, and the more negative conduction band potential offering more active species of ^1^O_2_ and ^•^O_2_^−^. Unexpectedly, the mu-CN/SiNSs-2 composite (m/u = 1/10) exhibited the highest activity for tetracycline antibiotic degradation, mainly due to the morphological advantage of a certain number of nanotubes generated on the CN/SiNSs hybrid nanosheets. It indicates that the supramolecular precursor strategy by synergistically using melamine and urea is highly efficient for the nanostructure construction of the CN/SiNSs hybrid system, enabling an appropriate nanostructure for the photodegradation of various pollutants.

## 1. Introduction

Water contamination caused by persistent organic pollutants (POPs, e.g., pesticides, dyes, and antibiotics) has raised widespread public concern due to the related health and safety problems. Advanced oxidation processes (AOPs) are appealing treatment options for the oxidation and mineralization of refractory and toxic POPs [1,2,3]. Traditional AOPs, such as ozone-, Fenton-, and persulfate-based systems, generate reactive oxygen species (ROS) via activating the stable precursors, including O_3_, H_2_O_2_, and peroxymonosulfate/peroxydisulfate. In particular, photocatalysis is an emerging AOP through the activation of O_2_ and/or H_2_O to yield ROS (such as hydroxyl radicals (^•^OH), superoxide radicals (^•^O_2_^−^), and singlet oxygen (^1^O_2_)) and holes (h^+^) under light radiation over photocatalysts. Graphitic carbon nitride (g-C_3_N_4_, CN) has been widely investigated as a semiconductor photocatalyst for POP purification [3], owing to the moderate band gap (~2.7 eV), appropriate band structure, good chemical and thermal stability, nontoxicity, cheapness, and easy synthesis [4]. However, the typical bulk CN faces many difficulties to achieve high photocatalytic activity, including small surface area, limited active sites, low visible-light utilization, and fast recombination of photogenerated carriers. Consequently, several strategies about the nano- and molecular-structure engineering of CN have been employed to overcome the weaknesses and improve the photocatalytic properties [5]. For example, the strategies of nanostructure construction are the most commonly used modification methods, since the CN nanostructures would supply larger surface areas, more active sites, easier access and shorter diffusion path for electrons, ions, and molecules, as well as the enlarged band gaps because of the quantum confinement effect of the nanostructures [6,7]. The supramolecular precursor strategy for CN nanostructure construction is a promising method that can produce g-C_3_N_4_ with an ordered texture and a controllable morphology [8], in which molecules are arranged into stable aggregates via non-covalent bond interactions under equilibrium conditions [9]. Hydrogen bonding is one of the most important interactions in arranging the structure of supramolecular aggregates because of its directionality and specificity [10]. In particular, the melamine precursor can connect with triazine derivatives (e.g., cyanuric acid) to form the melamine–cyanuric acid (MCA) supramolecular aggregates via hydrogen bonding, which can employ different linkages to form various morphologies, such as one-dimensional (1D) nanotubes [11,12], two-dimensional (2D) nanosheets [13,14], and 3D hollow spheres [15].

Moreover, various matrixes (e.g., oxides [16,17,18], clays [19,20,21], and carbon materials [22,23]) have been introduced to synthesize g-C_3_N_4_-based nanocomposites for overcoming their disadvantages of easy agglomeration, hard recovery, and high hydrophobicity. As one of the most commonly used matrixes, nanosilica has attracted considerable attention, particularly 2D silica nanosheets that possess a large surface/volume ratio and rich active sites. Our previous studies [24,25,26,27] have reported novel hierarchical porous silica nanosheets (SiNSs, Si_2_O_3_(OH)_2_) derived from natural kaolinite clay mineral. Compared to the general nanosilica (e.g., mesoporous silica), SiNSs own much more accessible hydroxyls [27,28], and they themselves are Janus nanosheets [29] made up of two different surfaces: a hydrophobic siloxane surface and a hydrophilic hydroxylated surface [30], and then this highly anisotropic shape permits the excellent emulsifying capacity and interfacial activity [31]. Therefore, SiNSs have been introduced as a supporter to assemble the highly dispersed O-doped g-C_3_N_4_ (OCN) nanoparticles though the thermopolymerization approach using precursor melamine in the sealed crucible, yielding the 0D/2D OCN/SiNSs composite [27]. However, the content of highly active OCN nanoparticles was low (16.5 wt%), and the morphology and structure significantly changed with increasing CN content; therefore, the unit activity of the CN nanostructure sharply reduced. Hence, a new strategy towards nanostructure construction needs to be proposed to simultaneously advance the content and unit activity of CN nanostructures, enabling higher photocatalytic activity, for example, the abovementioned MCA supramolecular precursor strategy.

Herein, we utilize the supramolecular precursor strategy of introducing MCA by synergistically using micromolecular precursors melamine (m) and urea (u) to further improve the photocatalytic activity of the CN/SiNSs hybrid nanosystem (mu-CN/SiNSs). The effect of the supramolecular precursor strategy of using diverse m/u mass ratios on the morphology of the CN nanostructure, and the corresponding microstructure, texture, optical property, electronic band structure, and the photocatalytic activity and mechanism for dye and antibiotic pollutants degradation, are researched in detail.

## 2. Materials and Methods

### 2.1. Materials

A high-purity kaolinite (A1_2_Si_2_O_5_(OH)_4_, >95%) clay mineral was acquired from China Kaolin Clay Co., Ltd. (Suzhou, China). Melamine (C_3_H_6_N_6_, ≥99%), urea (H_2_NCONH_2_, ≥99%), methanol (CH_3_OH, ≥99.5%), ethanol (C_2_H_5_OH, ≥99.7%), and Rhodamine B (C_28_H_31_ClN_2_O_3_, AR) were purchased from Sinopharm Chemical Reagent Co., Ltd. (Shanghai, China). Tetracycline hydrochloride (C_22_H_24_N_2_O_8_·HCl, 96%) was purchased from Shanghai Aladdin Biochemical Technology Co., Ltd. (Shanghai, China). Silica nanosheets (SiNSs) were produced by the thermal activation of layered kaolinite followed by selective etching-assisted exfoliation, according to previous work [27,30].

### 2.2. Preparation of g-C_3_N_4_/Silica Hybrid Nanosheets

As a control, three pure carbon nitrides (CNs), denoted as m-CN, u-CN, and mu-CN, were synthesized by the thermopolymerization method using melamine (m, 0.5 g), urea (u, 5 g), and a mixture of melamine and urea (0.25/2.5 g) as precursors, respectively. The precursors were added to a corundum crucible (30 mL) with four U-shaped windows (2 mm) and an inner-convex cover, and then calcined at 550 °C for 2 h in air atmosphere at a ramping rate of 5 °C min^−1^. As a result, the yields of m-CN, u-CN, and mu-CN were 0.153, 0.183, and 0.173 g, respectively.

To study the effects of the melamine–cyanuric acid (MCA) supramolecular precursor strategy by synergistically using the micromolecular precursors of melamine and urea on the structure tuning of g-C_3_N_4_, three mu-CN/SiNSs (50 wt% g-C_3_N_4_) composites were prepared as follows. The dosages of melamine and urea were 0.375/1.25 g, 0.25/2.5 g, and 0.125/3.75 g, respectively, denoted as mu-CN/SiNSs-1, mu-CN/SiNSs-2, and mu-CN/SiNSs-3 (mass ratio of m/u = 3/10, 1/10, and 1/30). The corresponding precursors were mixed beforehand with 0.25 g of SiNSs, and fully ground after adding 2 mL of methanol, dried at 60 °C for 1 h, and then placed into the abovementioned crucible, calcined at 550 °C for 2 h. Following several washes with deionized water and ethanol, the products were vacuum dried for 48 h at 30 °C. As a contrast, the m-CN/SiNSs and u-CN/SiNSs nanocomposites were prepared using the above method.

### 2.3. Characterization

A Rigaku MiniFlex-600 diffractometer with Cu Kα radiation operating at 40 kV and 15 mA was used to acquire X-ray diffraction (XRD) patterns. The Nicolet iS50 infrared spectrometer was used to determine Fourier-transform infrared (FTIR) spectra at a spectral resolution of 4 cm^−1^. Thermogravimetric (TG) analysis was performed in pure nitrogen with a heating rate of 5 °C min^−1^ on the NETZSCH STA 449 F5 Jupiter simultaneous thermal analyzer. N_2_ adsorption measurements were carried out on a Micromeritics ASAP 2460 instrument at 77 K, and the samples were degassed at 200 °C for more than 6 h. The specific surface areas (*S*_BET_) were calculated by the multi-point Brunauer–Emmett–Teller (BET) method; the total pore volumes (*V*_tot_) were estimated from the adsorbed volumes at a relative pressure of 0.992; the corresponding pore-size distributions were determined from the adsorption branches of the isotherms via the Barrett–Joyner–Halenda (BJH) method; and the micropore surface areas (*S*_micro_) and volumes (*V*_micro_) were obtained via the t-plot method. Scanning electron microscopy (SEM) was performed on a Hitachi Regulus 8220 instrument at an accelerating voltage of 3 kV, the samples were dispersed in ethanol by ultrasound for 20 min, deposited on copper foils, and sprayed with platinum to improve electrical conductivity. The K-Alpha^+^ spectrometer (Thermo Scientific, Waltham, MA, USA) with Al Kα monochromatic radiation was used for X-ray photoelectron spectroscopy (XPS) analysis after a surface Ar^+^ etching at 2 keV for 30 s. Binding energies (BE) of the XPS spectra were referenced to the C 1*s* peak of adventitious carbon at 284.8 eV. The Hitachi UH-4150 spectrophotometer with an integrating sphere system was used to measure UV-vis diffuse reflection spectra (DRS). Photoluminescence (PL) spectra were recorded on an Edinburgh FLS1000 photoluminescence spectrometer. The time-resolved PL (TRPL) spectra were performed on the Edinburgh FLS1000 fluorescence spectrometer with a 365 nm picosecond pulsed laser. Solid state nuclear magnetic resonance (SS-NMR) measurements were performed on a 600 MHz NMR spectrometer (JEOL ECZ600R/S3) equipped with a 14.09 T superconducting magnet and a 3.2 mm double resonance MAS probe.

### 2.4. Photocatalytic Degradation Experiments

The photocatalytic degradation experiments of RhB dye and TC antibiotic over the as-prepared photocatalysts were performed using a 300 W Xe lamp (PLS-SXE300+, Perfect Light) equipped with a 420 nm cut-off filter as the visible light source, and the lamp was placed 20 cm higher than the reaction solution in a 250 mL water-jacketed reactor coupled with a circulating cooling water system set at 20 °C. For a batch of degradation, 50 mg of photocatalyst was added into the water-jacketed reactor containing 100 mL of RhB (or TC) solution (10 mg L^−1^). After the adsorption in the dark for 30 min, the suspension was irradiated under visible light. At set intervals, 3 mL of the reaction solution was sampled and filtered by centrifugation (12,000 r/min). The corresponding RhB or TC absorbances were determined at λ_max_ = 554 or 357 nm by the Hitachi U-3900H spectrophotometer, respectively.

## 3. Results and Discussion

### 3.1. Nanocomposite Preparation

**Figure 1:**
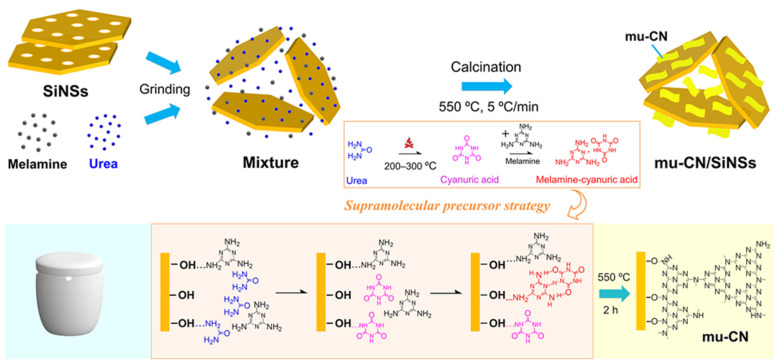
Schematic illustration of the mu-CN/SiNSs composite syntheses.

The schematic illustration in Figure 1 shows the supramolecular precursor strategy of preparing g-C_3_N_4_/silica hybrid nanosheets (mu-CN/SiNSs) via thermopolymerization by synergistically using the micromolecular precursors of melamine (m) and urea (u). The introduction of the melamine–cyanuric acid (MCA) supramolecular precursor through the thermal reaction between urea and melamine for the nanostructure construction of g-C_3_N_4_ on the SiNSs surface caused the effective tailoring of the features, including morphology and texture. Figure 1 shows that the micromolecular precursors of melamine and urea were uniformly mixed with SiNSs after fully grinding. During the thermopolymerization process under calcination, the periodic stacking of π-conjugated aromatic planes into layers was prevented due to the confinement effects of meso/macropores and hydroxyls of SiNSs, obtaining the CN nanosheets attached to SiNSs. The excessive melamine in the mixture (m/u = 3/10) for preparing mu-CN/SiNSs-1 would mainly generate the CN nanoparticles instead of nanosheets, similar to the m-CN/SiNSs sample. As for the mu-CN/SiNSs-2 sample (m/u = 1/10), a few CN nanotubes were constructed from the nanosheets, similar to the pure mu-CN sample (m/u = 1/10). Simultaneously, on the molecular scale, melamine and urea molecules were adsorbed by the hydroxyls and micropores on the hydroxylated surface of SiNSs, thus connecting with SiNSs via hydrogen bonds (−OH…NH_2_−). During heating up to 550 °C, the urea first pyrolyzed into cyanuric acid (CA) at 200–300 °C, and subsequently reacted with melamine to form the melamine–cyanuric acid (MCA) supramolecular aggregates via hydrogen bonds. Then, during the thermal polymerization process at 550 °C for 2 h, the MCA supramolecular aggregates combined with the remaining melamine or the CA micromolecular polymerized into an ordered CN nanosheet (main), except that the melamine was excessive (mu-CN/SiNSs-1), as mentioned above. Simultaneously, ammonia released from the Si−OH…NH_2_− groups between the CN precursors and SiNSs, thus the synthetized CN linked with SiNSs through Si−O−N bonds, but a few O atoms from Si−OH were doped into the heptazine (tri-s-triazine) units by substituting N atoms. Most notably, to avoid the great O-doping occurring in a hermetic environment [27], a corundum crucible with four U-shaped windows (Figure 1) was used in all preparations. Finally, the mass ratio of m/u had a great influence on the morphology of the CN nanostructure, as well as the corresponding texture, optical property, and electronic band structure.

### 3.2. Composition, Texture, and Morphology Characteristics

**Figure 2:**
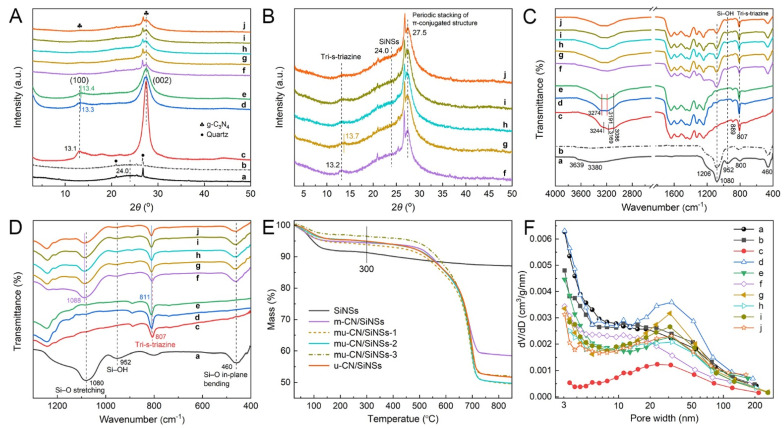
(**A**) Overall and (**B**) local enlarged XRD patterns; (**C**) overall and (**D**) local enlarged FTIR spectra; (**E**) TG curves; and (**F**) BJH pore-size distribution curves of (**a**) SiNSs, (**b**) SiNSs-550, (**c**) m-CN, (**d**) mu-CN, (**e**) u-CN, (**f**) m-CN/SiNSs, (**g**) mu-CN/SiNSs-1, (**h**) mu-CN/SiNSs-2, (**i**) mu-CN/SiNSs-3, and (**j**) u-CN/SiNSs.

XRD patterns of the as-prepared CNs and CN/SiNSs composites are displayed in Figure 2A. As a contrast, SiNSs exhibit a broad diffraction band assigned to SiO_4_ tetrahedral sheets at 2*θ* ≈ 24.0°, associated with trace impurities of quartz from the as-received kaolinite [24,25]. The diffraction reflections of m-CN (that is, bulk CN) are observed at about 13.1° and 27.5°, belonging to the typical heptazine units ((100) plane) and the periodic stacking of π-conjugated structures ((002) plane), respectively. The mu-CN and u-CN samples show similar patterns to m-CN, except that their (100) peaks shift from 13.1° to 13.3° and 13.4°, respectively, and the full width at the half maximum of (002) peaks obviously widen, demonstrating that the crystallinity of mu-CN and u-CN reduces due to the small size effect of their CN nanostructures. As for the CN/SiNSs composites, their widened (002) peaks also testify to the existence of CN nanostructures, and the close observation in Figure 2B shows the broad diffraction reflection of SiNSs at 24.0°. In addition, the (002) peaks of CN/SiNSs composites all remain at about 27.5°, corresponding to the same interplanar stacking distance of 0.324 nm, and their (100) peaks remain at about 13.2°, except for mu-CN/SiNSs-1 at 13.7° (Figure 2B).

FTIR spectroscopy is further measured to confirm the bonding structures of the mu-CN/SiNSs composites. Figure 2C shows the characteristic peaks of SiNSs at 1080, 800, and 460 cm^−1^ (SiO_4_ tetrahedra), 1206 and 1080 cm^−1^ (Si−O vibration), 3639 and 952 cm^−1^ (Si−OH bonds), and 3380 cm^−1^ (water OH vibration) [26,30]. The FTIR spectra of m-CN, mu-CN, and u-CN display the characteristic bands of g-C_3_N_4_ at about 807 and 1100−1800 cm^−1^, belonging to the breathing vibration of heptazine motifs and the skeletal vibrations of aromatic CN heterocycles [32], respectively. A broad band assigned to the N−H and O−H bonds of heptazine is observed at 2800−3500 cm^−1^ [33]. In particular, the characteristic peak of heptazine in mu-CN is evidently stronger than in m-CN and u-CN, indicating the more ordered g-C_3_N_4_ structure of mu-CN. Furthermore, the weaker bands of the N−H and O−H bonds in mu-CN and u-CN than in m-CN (bulk CN) confirm the increased crystallinity of the mu-CN and u-CN nanostructures compared to that of the bulk CN. The CN/SiNSs composites all exhibit the characteristic peaks attributed to SiNSs and g-C_3_N_4_. Close observation in the enlarged FTIR spectra (Figure 2D) shows that the band of Si−O stretching in CN/SiNSs composites shifts to 1088 from 1080 cm^−1^ (SiNSs), and the Si−OH peaks at 950 cm^−1^ are significantly weakened compared with SiNSs, possibly due to the generation of Si−O−N bonds between the SiNSs and g-C_3_N_4_ originating from the Si−OH…NH_2_− groups. Moreover, close observation of Figure 2D also shows that the peak of heptazine units is located at 807 cm^−1^ for m-CN and m-CN/SiNSs, and at 811 cm^−1^ for mu-CN, u-CN, mu-CN/SiNSs-1, 2, 3, and u-CN/SiNSs. It is seen that the urea-containing precursor-derived samples have a positive-shifted heptazine peak compared with the melamine precursor-derived samples, similar to the XRD results, probably because of the different chemical environments of heptazine units, which is also proven by the positive-shifted peaks of the N−H and O−H bonds at 2800−3500 cm^−1^ (Figure 2C). In brief, the above XRD and FTIR results verify that the emerging mu-CN and mu-CN/SiNSs composites consist of similar heptazine motifs to g-C_3_N_4_.

To determine the g-C_3_N_4_ content of the mu-CN/SiNSs composites, TG analysis (Figure 2E) was implemented in an N_2_ atmosphere. Pure SiNSs only showed an obvious mass loss in the range of 30−300 °C, mainly belonging to the absorbed water. All of the CN/SiNSs composites displayed two mass loss regions of the desorption of absorbed water (30−300 °C) and the decomposition of g-C_3_N_4_ (300−750 °C). Thus, excluding the absorbed water, the actual g-C_3_N_4_ content of the CN/SiNSs composites can be calculated according to the later mass loss region at 300−750 °C [27,34]. The corresponding contents of g-C_3_N_4_ in the m-CN/SiNSs, mu-CN/SiNSs-1, 2, 3, and u-CN/SiNSs composites are 37.3, 46.2, 46.7, 45.4, and 44.6 wt%, respectively. It is seen that the actual g-C_3_N_4_ contents of the mu-CN/SiNSs composites are all close to the theoretical value of 50 wt%.

The textural characteristics of photocatalysts including the specific surface area and porosity play an important role in photocatalytic reactions. Figure S1 and Figure 2F exhibit the N_2_ adsorption–desorption isotherms and pore-size distribution curves, respectively. Table 1 lists the related textural properties. From Table 1, SiNSs hold a large enough surface area (317 m^2^ g^−1^) and pore volume (0.38 cm^3^ g^−1^), showing the great potential as a carrier to disperse the g-C_3_N_4_ nanostructure. After calcination at 550 °C, SiNSs-550 shows a significantly reduced surface area of 144 m^2^ g^−1^ and a nearly unchanged pore volume of 0.32 cm^3^ g^−1^, which may be due to the surface micropore closure along with dehydroxylation, judging from the significant decrease of the micropore surface area (*S*_micro_) from 265 to 96 m^2^ g^−1^. The mu-CN sample holds a larger surface area (69 m^2^ g^−1^) and pore volume (0.36 cm^3^ g^−1^) than those of m-CN and u-CN, indicating the advantage of the supramolecular precursor strategy for CN nanostructure construction. The mu-CN/SiNSs composites show similar surface area and pore volume of about 65 m^2^ g^−1^ and 0.25 cm^3^ g^−1^, which are comparable to those of m-CN/SiNSs and u-CN/SiNSs (Table 1). N_2_ adsorption–desorption isotherms (Figure S1) show that all of the samples conform to a type IIB isotherm with a H3-type hysteresis loop for the sheet-like particles [26,35], representing the existence of meso/macropores. In addition, the rapid increase of adsorbed volume at P/P_0_ < 0.01 displays a large number of micropores in SiNSs, leading to the high surface area. However, the micropores are markedly reduced due to calcination for SiNSs-550. Therefore, the surface areas of the CN/SiNSs composites (Table 1) significantly decreased due to the decreased micropores of the SiNSs matrix, which are blocked by the synthesized CN nanostructures. In addition, the pore-size distribution curves (Figure 2F) display the rich meso/macropores of SiNSs ranging from 3 to 200 nm, and the calcination at 550 °C has almost no influence on the SiNSs-550, therefore providing enough confinement space for CN nanostructure construction. The mu-CN sample possesses a significantly larger volume of meso/macropores (3−200 nm) than m-CN and u-CN, leading to the larger surface area and pore volume, as mentioned above. It indicates that the supramolecular precursor strategy of synergistically using melamine and urea, along with the meso/macropores of SiNSs, are in favor of CN nanostructure construction. Therefore, the resultant mu-CN/SiNSs composites, especially mu-CN/SiNSs-1, 3, exhibit richer meso/macropores than m-CN/SiNSs and u-CN/SiNSs. It is noted that the m-CN/SiNSs sample has more mesopores ranging from 3 to 20 nm, explaining its high surface area of 86 m^2^ g^−1^. In brief, the mu-CN/SiNSs composites show a hierarchically porous structure with a large surface area and porosity, which would expose more active sites for adsorption and catalysis, and improve the reaction kinetics by enhancing the diffusion and transfer of reactants and products.

**Figure 3:**
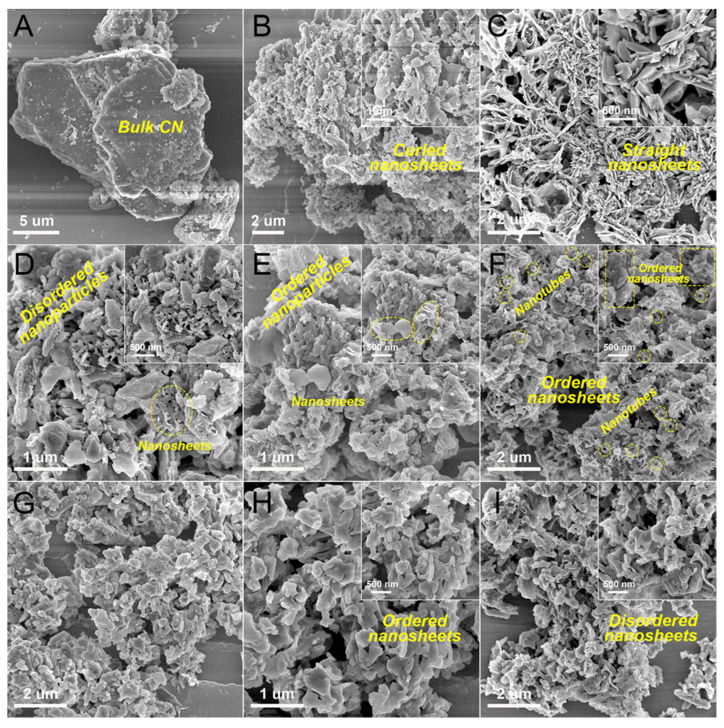
SEM images of (**A**) m-CN, (**B**) u-CN, (**C**) mu-CN, (**D**) m-CN/SiNSs, (**E**) mu-CN/SiNSs-1, (**F**) mu-CN/SiNSs-2, (**G**,**H**) mu-CN/SiNSs-3, and (**I**) u-CN/SiNSs.

The morphology of the mu-CN/SiNSs composites were observed by the SEM characterization (Figure 3). As a reference, Figure S2A displays the typical SEM image of SiNSs, showing the pseudo-hexagonal nanosheets (200−500 nm) originating from kaolinite [24]. After calcination at 550 °C, the nanosheet structure and porosity of SiNSs-550 showed almost no change (Figure S2B), conforming to the textural properties shown in Table 1. In Figure 3A, m-CN exhibited the bulk CN agglomerates with a layered structure, and u-CN showed the agglomerate of small curled nanosheets with quite a disordered structure (Figure 3B). Conversely, the mu-CN displayed the agglomerate of small straight nanosheets with an ordered structure (Figure 3C). Notably, as shown in the SEM images of the mu-CN powder without dispersing in ethanol by ultrasound (Figure S2C,D), some CN nanotubes exist on the nanosheet aggregates, indicating that the ordered nanosheets trend towards the generation of nanotubes, such as the generation of halloysite nanotubes from kaolinite nanosheets. The SEM images of m-CN/SiNSs and mu-CN/SiNSs-1 (Figure 3D,E) respectively show that the SiNSs were wrapped by the disordered and ordered CN nanoparticles at <100 nm, along with a small amount of CN nanosheets at <500 nm. However, when decreasing the m/u mass ratio, for mu-CN/SiNSs-2 (Figure 3F), the SiNSs are fully wrapped by the small and ordered CN nanosheets, and a little nanotube; as for mu-CN/SiNSs-3 (Figure 3G,H), particularly, it is seen that the agglomerates of large and ordered CN nanosheets tightly wrap the SiNSs. As a control, the SEM images of u-CN/SiNSs (Figure 3I) display that the large and disordered CN nanosheets wrap the SiNSs. To sum up, the supramolecular precursor strategy of introducing MCA by synergistically using melamine and urea can effectively promote the generation of ordered CN nanostructures, and the composition of m/u derived m/MCA/CA precursors decide their morphologies. Specifically, in the preparation of mu-CN/SiNSs composites, a high ratio of m/MCA resulted in ordered CN nanoparticles; a low ratio of MCA/CA led to small and ordered CN nanosheets, and a little nanotube; and high ratio of MCA/CA produced large and ordered CN nanosheets.

### 3.3. Surface Chemical State and Structural Characteristics

**Figure 4:**
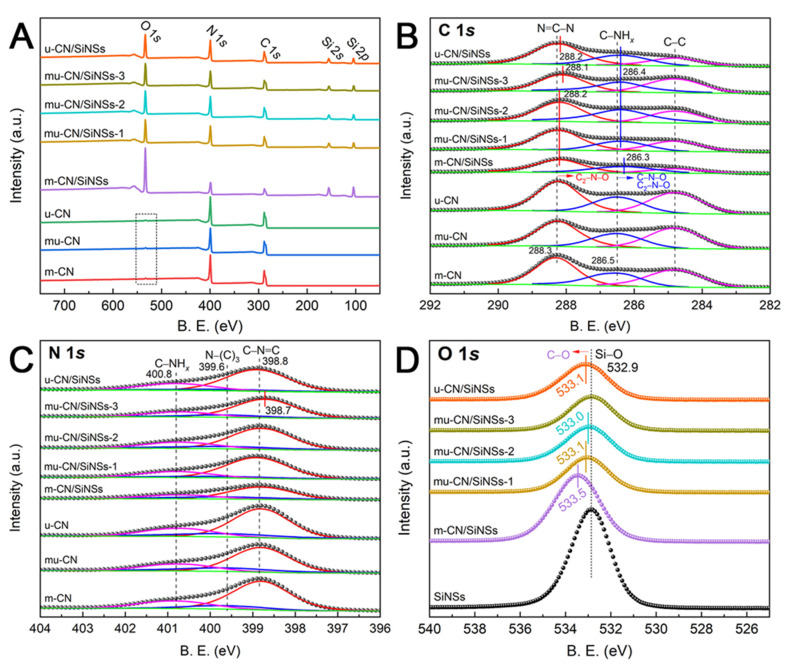
(**A**) XPS survey spectra and high-resolution (**B**) C 1*s*, (**C**) N 1*s*, and (**D**) O 1*s* spectra after Ar^+^ etching.

The surface elemental compositions and chemical states of the mu-CN/SiNSs composites are investigated by the XPS analysis, as shown in Table 2 and Figure 4. First of all, the existence of C, N, O, and Si elementals in the CN/SiNSs composites are testified by the surface elemental compositions and the survey XPS spectra in Figure 4A. Three CNs show very weak O 1*s* peaks with a similar O content of about 1 at% (Table 2), which is lower than that of our previously reported oxygen-doped carbon nitride (OCN, 5.97 at%). Notably, considering that the CN nanostructures are fully coated on the SiNSs surface (Figure 3), the surface elemental compositions after Ar^+^ etching can exactly reflect the changes of g-C_3_N_4_ in the CN/SiNSs composites. The atom ratios of N/C in mu-CN and u-CN are 1.40 and 1.48, which are higher than the value of 1.20 for m-CN, illustrating that the N-doping occurred during the polymerization process, possibly owing to the released NH_3_ from urea pyrolysis that doped into the CN framework [36]. Similarly, the mu-CN/SiNSs-1, 2 composites show higher N/C ratios than m-CN/SiNSs (1.21). However, it seems anomalous that mu-CN/SiNSs-3 and u-CN/SiNSs exhibit lower N/C ratios of 1.30 and 1.21, despite their higher urea dosage. This may be due to the significant release of NH_3_ from excess urea, which is not enough to react with CN framework. Furthermore, the O/Si atom ratios of CN/SiNSs composites are all equal to about 1.90, which is slightly lower than that of SiNSs (1.98), because of the decomposition of the Si−OH groups.

However, the C 1*s*, N 1*s*, and O 1*s* high-resolution XPS spectra demonstrated that the NH_3_-induced N-doping was too little to affect the chemical state. From Figure 4B, the C 1*s* spectra of m-CN, mu-CN, and u-CN all showed three peaks of C−C, C−NH*_x_*, and C−N=C at 284.8, 286.5, and 288.3 eV, respectively. Comparatively, the C−NH*_x_* peaks of CN/SiNSs composites shifted to low-binding energy (BE) at 286.4 eV, except for m-CN/SiNSs at 286.3 eV; meanwhile, their C−N=C peaks shifted to low BE at 288.2 eV, except for mu-CN/SiNSs-3 at 288.1 eV. These could be attributed to the link of the Si−O bonds with the N atoms of C−NH*_x_* and C−N=C, resulting in the generation of C−N−O and C_2_−N−O bonds at lower BE; thus, the SiNSs linked with CN through Si−O−N bonds (Figure 1). Moreover, the SiNSs preferentially linked with the N atoms of C−N=C for mu-CN/SiNSs-3, while this preferentially linked with the N atoms of C−NH*_x_* for m-CN/SiNSs. In Figure 4C, the N 1*s* spectra of the CNs and CN/SiNSs composites all displayed three peaks of C−N=C, N−(C)_3_, and C−NH*_x_* at about 398.8, 399.6, and 400.8 eV [7], respectively, except that the C−N=C peak of mu-CN/SiNSs-3 shifts to lower BE at 398.7 eV, which could be due to the preferential link of SiNSs with the N atoms of C−N=C for mu-CN/SiNSs-3, consistent with the C 1*s* spectrum. Moreover, the O 1*s* spectra in Figure 4D displayed the Si−O species at 532.9 eV for SiNSs. Except for mu-CN/SiNSs-3, all of the CN/SiNSs composites showed a shift to higher BE attributed to the formation of C−O species (e.g., C−O−C and C−O−H [37]) through O-doping by replacing the N atoms of heptazine. What is more, it seemed that the higher the melamine dosage, the more O-doping that occurred; therefore, m-CN/SiNSs had the maximum shift to 533.5 eV, even though the O-doping amount was still lower than our previously reported OCN/SiNSs composites [27], and thus could not obviously affect the C and N chemical states of CN (Figure 4B,C). In sum, the XPS analysis indicated that the SiNSs linked with CN through Si−O−N bonds, and the N, O-doping occurring in CN was too few to be noted; moreover, the mu-CN/SiNSs-3 composite showed the strongest interaction between SiNSs and the CN nanosheets.

**Figure 5:**
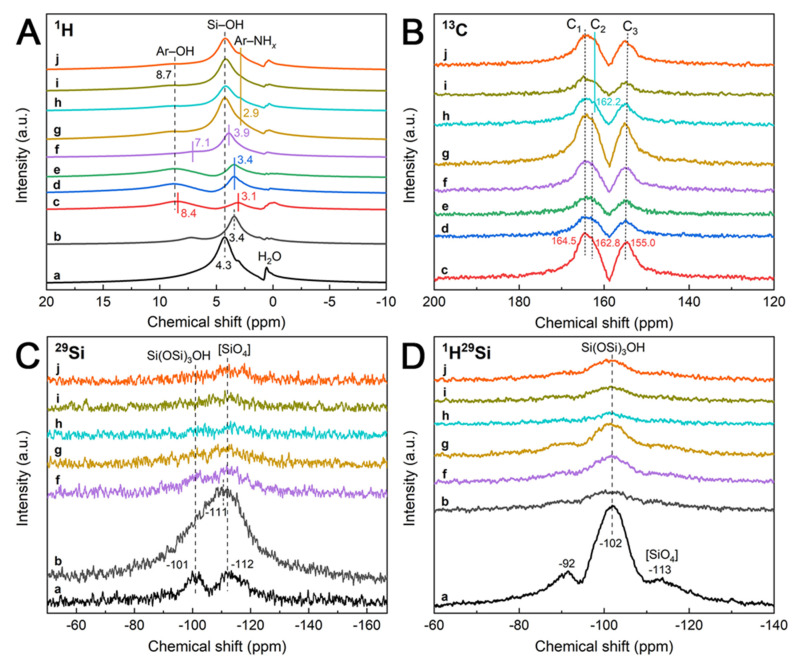
Solid-state (**A**) ^1^H, (**B**) ^13^C, (**C**) ^29^Si MAS, and (**D**) ^1^H^29^Si CP/MAS NMR spectra of (**a**) SiNSs, (**b**) SiNSs-550, (**c**) m-CN, (**d**) mu-CN, (**e**) u-CN, (**f**) m-CN/SiNSs, (**g**) mu-CN/SiNSs-1, (**h**) mu-CN/SiNSs-2, (**i**) mu-CN/SiNSs-3, and (**j**) u-CN/SiNSs.

The solid-state ^1^H, ^13^C, ^29^Si MAS, and ^1^H^29^Si CP/MAS NMR spectra are supplied to determine the structural characteristics. As shown in the ^1^H SS-NMR spectra (Figure 5A), SiNSs show a characteristic signal of Si−OH (Si(OSi)_3_OH) at δ = 4.3 ppm; after calcination, the Si−OH peak of SiNSs-550 shifts to 3.4 ppm due to the polymerization of Si(OSi)_3_OH units. The m-CN shows two characteristic signals of aromatic hydroxyl (Ar−OH) and aromatic amine (Ar−NH*_x_*) of g-C_3_N_4_ at δ = 8.4 and 3.1 ppm. However, for mu-CN and u-CN, the Ar−OH and Ar−NH*_x_* peaks shift to 8.7 and 3.4 ppm, respectively, due to their different surface chemical environments of heptazine units forming m-CN, which is consistent with the FTIR results of the surface N−H and O−H bonds of heptazine units (Figure 2C). Moreover, for the m-CN/SiNSs composite, the Ar−OH peak obviously shifts to 7.1 ppm, and the Si−OH and Ar−NH*_x_* show an overlapped signal at 3.9 ppm, probably due to the strong interaction between the Si−OH of SiNSs and the heptazine units of CN in m-CN/SiNSs. Contrarily, the mu-CN/SiNSs and u-CN/SiNSs composites all display similar signals to Ar−OH, Si−OH, and Ar−NH*_x_* at 8.7, 4.3, and 2.9 ppm, respectively. From the ^13^C SS-NMR spectra in Figure 5B, the CNs and CN/SiNSs composites all exhibit three characteristic signals of C−NH_2_ (C_1_), C−NH (C_2_), and CN_3_ (C_3_) of the heptazine units at about δ = 164.5, 162.8, and 155.0 ppm [37], respectively, except that the C_2_ peaks of mu-CN/SiNSs-2, 3 and u-CN/SiNSs shift to 162.2 ppm, indicating that the abundant urea precursor leads to the strong interaction between the Si−OH of SiNSs and the C−NH of heptazine.

From the ^29^Si MAS SS-NMR spectra in Figure 5C, the SiNSs show two signals of the Si(OSi)_3_OH unit (Q^3^) and SiO_4_ tetrahedral structure unit (Q^4^) at δ = −101 and −112 ppm; after calcination, the SiNSs-550 exhibits an overlapped signal at −111 ppm, mainly belonging to the SiO_4_ tetrahedral structure unit. As for the CN/SiNSs composites, their ^29^Si signals are too weak to be clearly distinguished. Hence, the ^1^H^29^Si CP/MAS SS-NMR spectra (Figure 5D) are further offered to examine the structure of the CN/SiNSs composites. The SiNSs sample displays three signals of the layer structure unit (Q^3^), Si(OSi)_3_OH unit (Q^3^), and SiO_4_ tetrahedral structure unit (Q^4^) at δ = −92, −102, and −113 ppm, respectively [28]; after calcination, these three signals from SiNSs-550 are significantly weakened, similar to the CN/SiNSs composites. Moreover, the relative order of their signal strengths is mu-CN/SiNSs-1 > m-CN/SiNSs > u-CN/SiNSs > mu-CN/SiNSs-3 > mu-CN/SiNSs-2, and the higher the signal strength, the more Si(OSi)_3_OH units. In other words, the mu-CN/SiNSs-2, 3 nanocomposites with fewer Si(OSi)_3_OH units could exhibit stronger interactions between SiNSs and CN nanostructures. Therefore, the SS-NMR spectra confirm the XPS results (Figure 4), together revealing the high chemical activity of the mu-CN/SiNSs-2, 3 nanocomposites, especially mu-CN/SiNSs-3.

### 3.4. Photocatalytic Activity and Mechanism

#### 3.4.1. Photocatalytic Activity of mu-CN/SiNSs Nanocomposites

**Figure 6:**
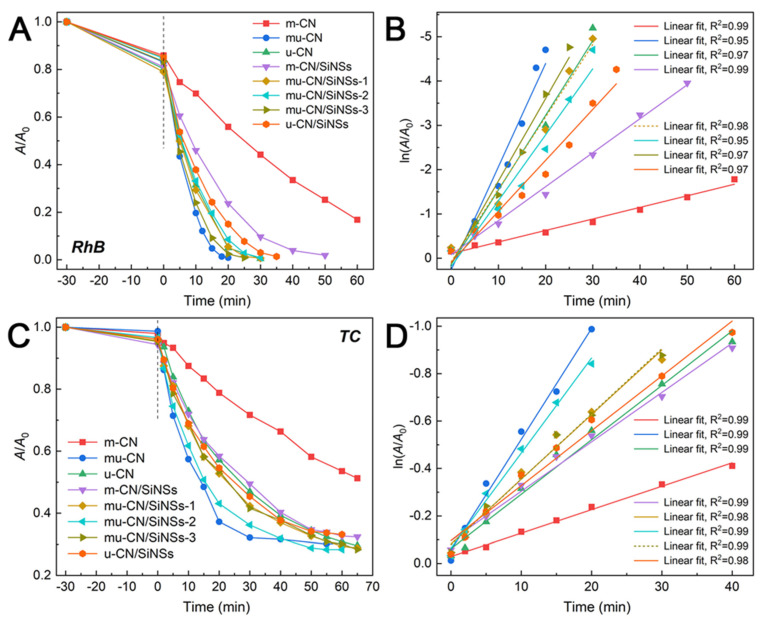
*A*/*A*_0_ and ln(*A*/*A*_0_) versus reaction time for the photocatalytic degradation of (**A**,**B**) RhB and (**C**,**D**) TC under visible-light irradiation over the nanocatalysts.

The photocatalytic properties of the mu-CN/SiNSs composites were evaluated by the degradation of typical Rhodamine B (RhB) dye and Tetracycline (TC) antibiotic pollutants. As shown in Figure 6A and Table 3, the RhB dye is almost completely degraded within 20, 30, 50, 30, 30, 25, and 35 min over the nanocatalysts of mu-CN, u-CN, m-CN/SiNSs, mu-CN/SiNSs-1, 2, 3, and u-CN/SiNSs, respectively. However, the m-CN sample, that is, bulk CN, is far from completely degrading RhB within 60 min. As shown in Figure 6B and Table 3, the relevant apparent kinetic rate constants (*k*_app_) of the photocatalysts are estimated using the following equation: ln(*A*/*A*_0_) = *k*_app_·*t*, where *A*_0_ and *A* represent the initial absorbance and the absorbance at time *t*. The *k*_app_ values of m-CN, mu-CN, u-CN, m-CN/SiNSs, mu-CN/SiNSs-1, 2, 3, and u-CN/SiNSs are 0.026, 0.233, 0.168, 0.077, 0.164, 0.149, 0.186, and 0.115 min^−1^, respectively. Thus, the mu-CN and mu-CN/SiNSs-3 composite have the highest catalytic activity among the CNs and CN/SiNSs composites, respectively, and their activities are very close. Moreover, as shown in Table S1, the *k*_app_ value of mu-CN/SiNSs-3 (0.186 min^−1^) is much higher than those of most other g-C_3_N_4_ based nanocomposites [38], including our previously reported OCN/SiNSs (0.066 min^−1^) [27], proving the advantage of the supramolecular precursor strategy in improving the photoreactivity of the CN/SiNSs hybrid nanosystem.

It is well known that the silica supporters are an inert material and a typical insulator. For this reason, as reported in previous work [27], the SiNSs showed a much smaller *k*_app_ value of 0.006 min^−1^ than g-C_3_N_4_. Therefore, the semiconductor g-C_3_N_4_ in the composites is mainly responsible for the enhanced photocatalytic activity. To study the unit activity of g-C_3_N_4_ in the composites, the *k*_app_ values are normalized with the mass of g-C_3_N_4_ to offer the *κ* values in Table 3. It is found that m-CN shows the minimum *κ* value (0.52 min^−1^ g^−1^), and the other CNs and CN/SiNSs composites with CN nanostructures have at least 6.5 times more value than m-CN. Moreover, the *κ* value of mu-CN is larger than u-CN, while those of the mu-CN/SiNSs-1, 2, 3 composites are higher than m-CN/SiNSs and u-CN/SiNSs, together indicating the effectiveness of the supramolecular precursor strategy in enhancing the nanostructure-dependent photoreactivity of g-C_3_N_4_. Meanwhile, the *κ* values of the mu-CN/SiNSs composites are still much higher than mu-CN, demonstrating the great contribution of the SiNSs matrix. In addition, the mu-CN/SiNSs-3 composite holds the maximum *κ* value of 8.19 min^−1^ g^−1^, which is 1.8 times higher than that of mu-CN (4.66 min^−1^ g^−1^), and then, considering the similar specific surface area and porosity of the mu-CN/SiNSs composites, their different morphologies of highly dispersed and ordered CN nanostructures (nanoparticle/tube/sheet), as shown in the SEM images (Figure 3E–H), decide their photoreactivity. Therefore, when decreasing the m/u mass ratio, the *κ* values of mu-CN/SiNSs-3 are obviously higher than mu-CN/SiNSs-1, 2 due to the ripeness of the ordered CN nanosheets, yielding typical g-C_3_N_4_/silica hybrid nanosheets.

On the other hand, the photocatalytic activities of the mu-CN/SiNSs composites towards TC antibiotic degradation are shown in Figure 6C,D, and the corresponding *k*_app_ and *κ* values are summarized in Table 4. It can be seen that the CNs and CN/SiNSs composites show a lower photocatalytic activity towards TC than that for RhB, probably due to their significantly lower adsorption towards TC than that for RhB (Table 3 and Table 4). Although mu-CN holds the highest *k*_app_ of 0.047 min^−1^, the mu-CN/SiNSs-2 composite exhibits a similar value of 0.040 min^−1^ and a higher TC degradation rate within 50 min (Figure S3). The analysis of the *κ* values of TC photodegradation also proves the great contribution of the highly dispersed and ordered CN nanostructures on the photoreactivity. However, this time, the mu-CN/SiNSs-2 composite has the maximum *κ* value (1.71 min^−1^ g^−1^), which is about 1.8 times higher than that of mu-CN (0.94 min^−1^ g^−1^). Different from the key role of the ordered CN nanosheets in RhB degradation, a certain amount of the ordered CN nanotubes existing in mu-CN and mu-CN/SiNSs-2 plays a pivotal role in TC degradation.

#### 3.4.2. Photoabsorption and Photogenerated Charge Carriers

**Figure 7:**
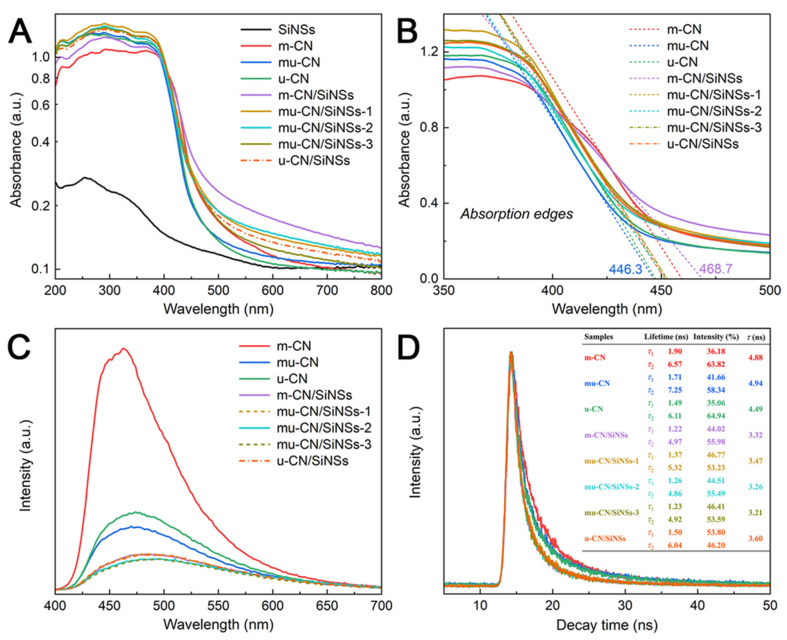
(**A**) UV-vis DRS spectra, (**B**) absorption edges, (**C**) photoluminescence (PL) spectra, and (**D**) time-resolved PL spectra of the photocatalysts.

To further clarify the structure–activity relationship, the photoabsorption properties of the mu-CN/SiNSs photocatalysts are investigated by UV-vis diffuse reflectance spectroscopy (DRS) in Figure 7A,B. From Figure 7A, the mu-CN and u-CN samples harvest more light at <420 nm and less visible light at >420 nm compared with m-CN, owing to the multiple reflection/scattering of light within the pores of the CN nanostructures and the quantum confinement effect of the CN nanostructures, respectively. Conversely, all of the CN/SiNSs composites absorb more visible light at 420−800 nm than m-CN, indicating that the CN nanostructures highly dispersed on the SiNSs substrate could exhibit higher visible light absorption, probably owing to the visible light reflection/scattering on SiNSs [27,39]. Among the composites, m-CN/SiNSs evidently shows the highest visible light absorption. To further explain the above photoabsorption properties, Figure 7B shows the absorption edges (λ) of m-CN, mu-CN, u-CN, m-CN/SiNSs, mu-CN/SiNSs-1, 2, 3, and u-CN/SiNSs, which are 459.3, 446.3, 447.2, 468.7, 452.5, 451.6, 452.3, and 451.4 nm, respectively. Moreover, the corresponding band gaps (E_g_) are determined by the absorption edges using the empirical formula E_g_ = 1240/λ [40], and the results are 2.70, 2.78, 2.77, 2.65, 2.74, 2.75, 2.74, and 2.75 eV, respectively. Except for m-CN/SiNSs, the E_g_ values of the CNs and CN/SiNSs composites all show a slight reduction relative to m-CN. According to the XPS results, the m-CN/SiNSs composite has relatively more O-doped CN than other samples; compared with m-CN, the quantum confinement effect of the CN nanoparticles in m-CN/SiNSs enlarges the band gap, while the O-doping narrows it; therefore, the comprehensive impacts on the electronic band structure results in the slightly narrowed band gap of m-CN/SiNSs, which permits m-CN/SiNSs to absorb more visible light (Figure 7A). Conversely, the other CNs and CN/SiNSs composites have the enlarged band gaps attributed to the single quantum confinement effect of CN nanostructures.

The photogenerated charge carriers’ separation efficiency of the mu-CN/SiNSs photocatalysts are proven by the PL spectra (Figure 7C) and the time-resolved PL (TRPL) decay spectra (Figure 7D). It is known that the lower intensity of the PL emission peak reflects the lower rate of the photogenerated electron–hole pair recombination. As shown in Figure 7C, the mu-CN and u-CN nanostructures exhibit the much weaker emission peaks than the bulk CN of m-CN; moreover, mu-CN is better than u-CN, indicating the contribution of the supramolecular precursor strategy to the separation of photogenerated charge carriers. In addition, the CN/SiNSs composites exhibit lower PL emission peak intensity than pure CNs, illustrating that the dispersion effect of the SiNSs matrix on CN nanostructures can further reduce the photoinduced carriers’ separation efficiency. Benefitting from both the supramolecular precursor strategy and the dispersion effect of the SiNSs matrix, the mu-CN/SiNSs-2, 3 composites display the lowest emission peak intensity. The TRPL decay spectra (Figure 7D) are further provided to study the photoinduced charge separation dynamics of the mu-CN/SiNSs composites. The shorter lifetime (τ_1_), longer lifetime (τ_2_), and their percent contributions, as well as the average lifetime (τ), are calculated by a double-exponential fitting. In comparison, the mu-CN/SiNSs-2, 3 composites show the minimum τ values of 3.26 and 3.21 ns, indicating their more efficient transfer of photoinduced charge carriers. Moreover, the shorter lifetime τ_1_ of intrinsic fluorescence is associated with the radiative decay from the excited state to the ground state [39]; thus, the low τ_1_ value and contribution reflect a low amount of rapidly recombined charge carriers for the mu-CN/SiNSs-2, 3 composites, owing to the better transfer pathway and shorter transfer distance of the electron–hole pairs for the highly dispersed CN nanosheet/tubes on the SiNSs surface [41].

#### 3.4.3. Band Structure and Semiconductor Photocatalytic Mechanism

**Figure 8:**
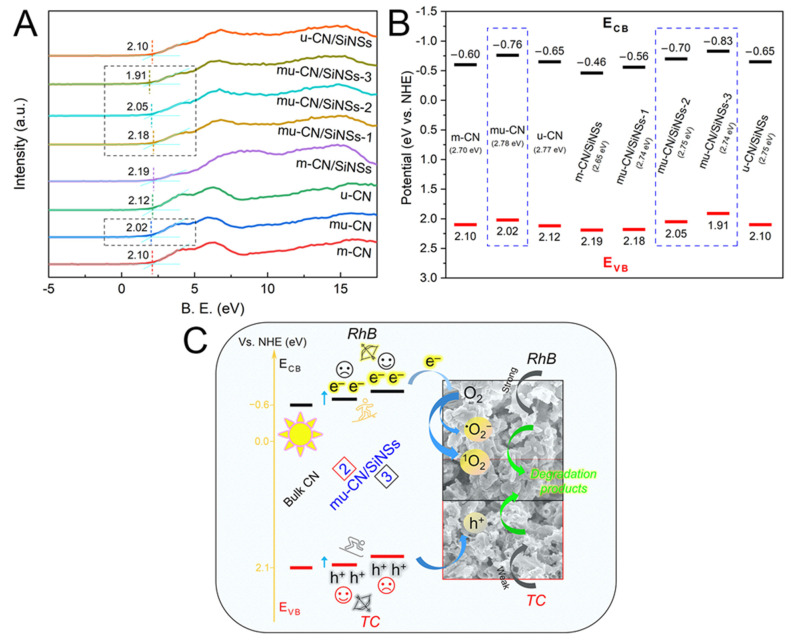
(**A**) VB-XPS spectra and (**B**) band structure of the CNs and CN/SiNSs composites; (**C**) schematic photocatalytic degradation mechanism of RhB and TC over the mu-CN/SiNSs-2, 3 composites.

To determine the electronic band structures of the mu-CN/SiNSs photocatalysts, the valence band (VB) XPS spectra are displayed in Figure 8A. The VB positions (E_VB_) of m-CN, mu-CN, u-CN, m-CN/SiNSs, mu-CN/SiNSs-1, 2, 3, and u-CN/SiNSs are recorded as 2.10, 2.02, 2.12, 2.19, 2.18, 2.05, 1.91, and 2.10 eV, respectively. According to the E_g_ values in the UV-vis DRS analysis, the relevant conduction band (CB) positions (E_CB_) are calculated to be −0.60, −0.76, −0.65, −0.46, −0.56, −0.70, −0.83, and −0.65 eV, respectively. Thus, the band structures of the CNs and CN/SiNSs composites are displayed in Figure 8B. As mentioned above, compared to the bulk CN of m-CN, the band gaps of mu-CN and u-CN could be enlarged by shifting VB and CB edges in opposite directions because of the quantum confinement effect. Meanwhile, O-doping by substituting within the N atoms of heptazine affects the CB potential; therefore, the comprehensive influences result in the less negative CB potential and more positive VB potential of m-CN/SiNSs and mu-CN/SiNSs-1 than those of m-CN. The u-CN and u-CN/SiNSs show the slightly higher VB and less CB potentials due to the oppositely shifting band edges (quantum confinement effect of CN nanosheets). As for the mu-CN and mu-CN/SiNSs-2, 3 composites, excluding the influences of O-doping and N-doping, their obviously lower VB and CB potentials could be due to their well-ordered CN nanosheets (Figure 3C,F,H), which lower both the VB and CB edges compared with the disordered CN nanosheets of u-CN and u-CN/SiNSs (Figure 3B,I). The more ordered the structure, the lower the VB and CB potentials, particularly in the mu-CN/SiNSs-3 composite.

The semiconductor mechanism has been widely studied to describe the photocatalytic process [3,42]. Generally, the excited-state properties of the g-C_3_N_4_ semiconductor (that is, the photogenerated electrons (e^−^) and holes (h^+^)), the later-generated reactive oxygen species (e.g., hydroxyl radicals (^•^OH), superoxide radicals (^•^O_2_^−^), and singlet oxygen (^1^O_2_)), and direct reaction pathways (e.g., oxidation of h^+^) with reactants are the critical factors that determine the photocatalytic activity. Different from the usual active species of h^+^ and ^•^O_2_^−^ reported for the g-C_3_N_4_-based photocatalysts, the CN/SiNSs hybrid nanosystem suggests the main active species of ^1^O_2_, ^•^O_2_^−^, and h^+^ [27]. Herein, according to the band structure in Figure 8B, the more negative CB potentials of −0.70, −0.77, and −0.83 eV for mu-CN/SiNSs-2, mu-CN, and mu-CN/SiNSs-3 yield more e^−^ to react with O_2_ in the CB edge, therefore producing a great quantity of active species of ^1^O_2_ (preferential) and ^•^O_2_^−^; meanwhile, the more positive VB potentials of 2.12, 2.18, and 2.19 eV for u-CN, mu-CN/SiNSs-1, and m-CN/SiNSs exhibit the stronger oxidability of h^+^ in the VB edge.

To summarize, the possible photocatalytic mechanism of the mu-CN/SiNSs composites can be described as follows. Although m-CN/SiNSs holds the larger surface area, higher visible light absorption, and similarly high photogenerated carrier separation efficiency compared to the mu-CN/SiNSs composites, it still shows the low unit activity of CN nanoparticles due to the disordered structure, and the much higher CB potential with poor reactive oxygen species. On the contrary, in addition to the large surface area, visible light absorption, and photogenerated carrier separation efficiency, the mu-CN/SiNSs composites also display ordered CN nanostructures with different morphologies of particle, tube, and sheet (Figure 3), in which the typical g-C_3_N_4_/silica hybrid nanosheets of mu-CN/SiNSs-3 with larger CN nanosheets exhibit the highest activity towards RhB photodegradation due to its most negative CB edge potential offering the most active oxygen species of ^1^O_2_ and ^•^O_2_^−^ under visible-light irradiation. Moreover, the mu-CN/SiNSs-2 composite with a certain amount of CN nanotubes generated on g-C_3_N_4_/silica hybrid nanosheets shows the highest activity towards TC antibiotic photodegradation, owing to the morphological advantage of nanotube in TC degradation compared to the nanoparticle/sheet, and the more positive VB edge potential providing a higher oxidability of h^+^ towards TC, as shown in Figure 8C. Moreover, the CN/SiNSs hybrid nanosystem has much stronger adsorption towards RhB than that for TC, leading to the much higher photocatalytic activity towards RhB dye.

## 4. Conclusions

In summary, we have successfully constructed the mu-CN/SiNSs composites via the MCA supramolecular precursor strategy by synergistically using melamine and urea. The results showed that the introduction of MCA supramolecular aggregates promoted the generation of ordered CN nanostructures (~50 wt%) on the SiNSs surface, and the morphology of the CN nanostructure (e.g., nanoparticle/tube/sheet) could be regulated through the mass ratio of m/u due to the different compositions of the m/u-derived m/MCA/CA precursors. When the m/u ratio is 1/30, a typical mu-CN/SiNSs-3 hybrid nanosheet was prepared, and displayed high photocatalytic activity for RhB degradation within 25 min with a *k*_app_ value of 0.186 min^−1^, which is much better than most other g-C_3_N_4_-based nanocomposites. This may be due to the large surface area of the highly dispersed and ordered CN nanosheets, the strong interaction between CN and SiNSs, the high photogenerated carrier separation efficiency, and the more negative CB potential providing more active species of ^1^O_2_ and ^•^O_2_^−^. Furthermore, the mu-CN/SiNSs-2 composite (m/u = 1/10) possessed the highest activity for TC antibiotic degradation, due to the morphological advantage of a certain number of nanotubes generated on the hybrid nanosheets, and the more positive VB potential providing higher oxidability of h^+^ towards TC. This study testifies to the great potential of the MCA supramolecular precursor strategy by using melamine/urea in constructing appropriate nanostructures of a CN/SiNSs hybrid system for photodegrading various pollutants.

## Figures and Tables

**Table 1:** Textural properties of the SiNSs, SiNSs-550, CNs, and CN/SiNSs composites.

Samples	*S*_BET_ (m^2^ g^−1^)	*S*_micro_^a^ (m^2^ g^−1^)	*V*_tot_ (cm^3^ g^−1^)	*V*_micro_^a^ (cm^3^ g^−1^)	*D*_Avg_ (nm)
SiNSs	317	265	0.38	0.11	5
SiNSs-550	144	96	0.32	0.04	9
m-CN	16	2	0.12	0.001	30
mu-CN	69	6	0.36	0.003	21
u-CN	49	6	0.25	0.003	20
m-CN/SiNSs	86	48	0.21	0.02	10
mu-CN/SiNSs-1	71	30	0.28	0.01	16
mu-CN/SiNSs-2	61	25	0.23	0.01	15
mu-CN/SiNSs-3	67	27	0.25	0.01	15
u-CN/SiNSs	70	35	0.26	0.02	15

**Table 2:** Surface elemental compositions of the SiNSs, CNs, and CN/SiNSs composites determined by XPS analysis.

Samples	Elemental Composition (at%)				N/C	O/Si
	C	N	O	Si		
SiNSs	4.46	0.56	63.14	31.84	–	1.98
m-CN	44.98	53.91	0.96	0.15	1.20	–
mu-CN	41.07	57.58	1.14	0.20	**1.40**	–
u-CN	39.80	58.97	1.10	0.13	**1.48**	–
m-CN/SiNSs	14.95	18.10	43.91	23.04	1.21	**1.91**
mu-CN/SiNSs-1	24.31	35.06	26.35	14.27	**1.44**	1.85
mu-CN/SiNSs-2	25.33	35.52	25.35	13.8	**1.40**	1.84
mu-CN/SiNSs-3	25.34	33	27.05	14.61	1.30	1.85
u-CN/SiNSs	28.09	34.05	24.55	13.31	1.21	1.84

**Table 3:** Photocatalytic performance of the CNs and CN/SiNSs composites towards RhB degradation.

Catalyst	g-C_3_N_4_ Content (wt%)	*t* (min)	Adsorption ^a^ (%)	*k*_app_ (min^−1^)	*κ* ^b^ (min^−1^ g^−1^)
m-CN	100	>60	14	0.026	0.52
**mu-CN**	100	20	17	**0.233**	4.66
u-CN	100	30	20	0.168	3.36
m-CN/SiNSs	37.3	50	19	0.077	4.13
mu-CN/SiNSs-1	46.2	30	21	0.164	7.10
mu-CN/SiNSs-2	46.7	30	15	0.149	6.38
**mu-CN/SiNSs-3**	45.4	25	17	**0.186**	**8.19**
u-CN/SiNSs	44.6	35	15	0.115	5.16

^a^ RhB adsorption rates over the photocatalysts in dark. ^b^ *κ*: apparent rate constant per unit mass of g-C_3_N_4_, min^−1^ g^−1^.

**Table 4:** Photocatalytic performance of the CNs and CN/SiNSs composites towards TC degradation.

Catalyst	Adsorption (%)	*k*_app_ (min^−1^)	*κ* (min^−1^ g^−1^)
m-CN	2	0.010	0.20
**mu-CN**	1	**0.047**	0.94
u-CN	3	0.023	0.46
m-CN/SiNSs	6	0.021	1.13
mu-CN/SiNSs-1	4	0.027	1.17
**mu-CN/SiNSs-2**	3	**0.040**	**1.71**
mu-CN/SiNSs-3	5	0.028	1.23
u-CN/SiNSs	4	0.023	1.03

## Supplementary Materials

The following supporting information can be downloaded at: https://www.mdpi.com/article/10.3390/nano12183108/s1, Table S1: Comparison of photocatalytic performances; Figure S1: N_2_ adsorption–desorption isotherms; Figure S2: SEM images of SiNSs, SiNSs-550, and um-CN; Figure S3: Degradation rates of RhB and TC over the photocatalysts.
