# Supplementary material for: Supramolecular Precursor Strategy to Construct g-C3N4/Silica Hybrid Nanosheets for Photocatalytic Degradation of Dye and Antibiotic Pollutants
Source: Nanomaterials (Basel). 2022 Sep 7;12(18):3108. doi: 10.3390/nano12183108 (PMC9503609; doi:10.3390/nano12183108)
Supplement: Supplementary file 1 [file nanomaterials-12-03108-s001.zip › nanomaterials-1894211-supplementary.pdf]

Supporting Information

Supramolecular Precursor Strategy to Construct g-C_3N_4/Silica Hybrid Nanosheets for Photocatalytic Degradation of Dye and Antibiotic Pollutants

Yongsheng Yu [1], Jinghan Wang [1], Zhaoli Yan [1,*], Qiangshan Jing [1], Peng Liu [1,*] and Bing Xu [2]

[1] *Henan Province Key Laboratory of Utilization of Non-Metallic Mineral in the South of Henan, College of Chemistry and Chemical Engineering, Xinyang Normal University, Xinyang 464000, China*

[2] *School of Chemistry and Chemical Engineering, Henan Polytechnic University, Jiaozuo 454000, China*

Author Information

Corresponding Author

* Email: yanzhaoli86@163.com, zhaoliyan@xynu.edu.cn (Z.Y.), liupeng1621@163.com (P.L.);

Tel./Fax: +86-376-6390603

Table S1. Comparison of photocatalytic performances of the um-CN/SiNSs composite and other g-C_3N_4 based nanocomposites towards RhB.

Catalyst	g-C_3N_4 content (wt%)	t (min)	k_{app} (min^{-1})
kaolinite/g-C_3N_4 [1]	28.6	360	–
g-C_3N_4/SiO_2 [2]	95	100	0.023
h-BN/g-C_3N_4 [3]	–	40	0.131
N-CNT/mpg-C_3N_4 [4]	–	60	–
OMC/g-C_3N_4 [5]	–	60	0.053
1.5OCN/SiNSs [6]	52.5	60	0.066
MCA-CN [7]	100	60	0.062
**mu-CN**	**100**	**20**	**0.233**
**mu-CN/SiNSs-3**	**45.4**	**25**	**0.186**

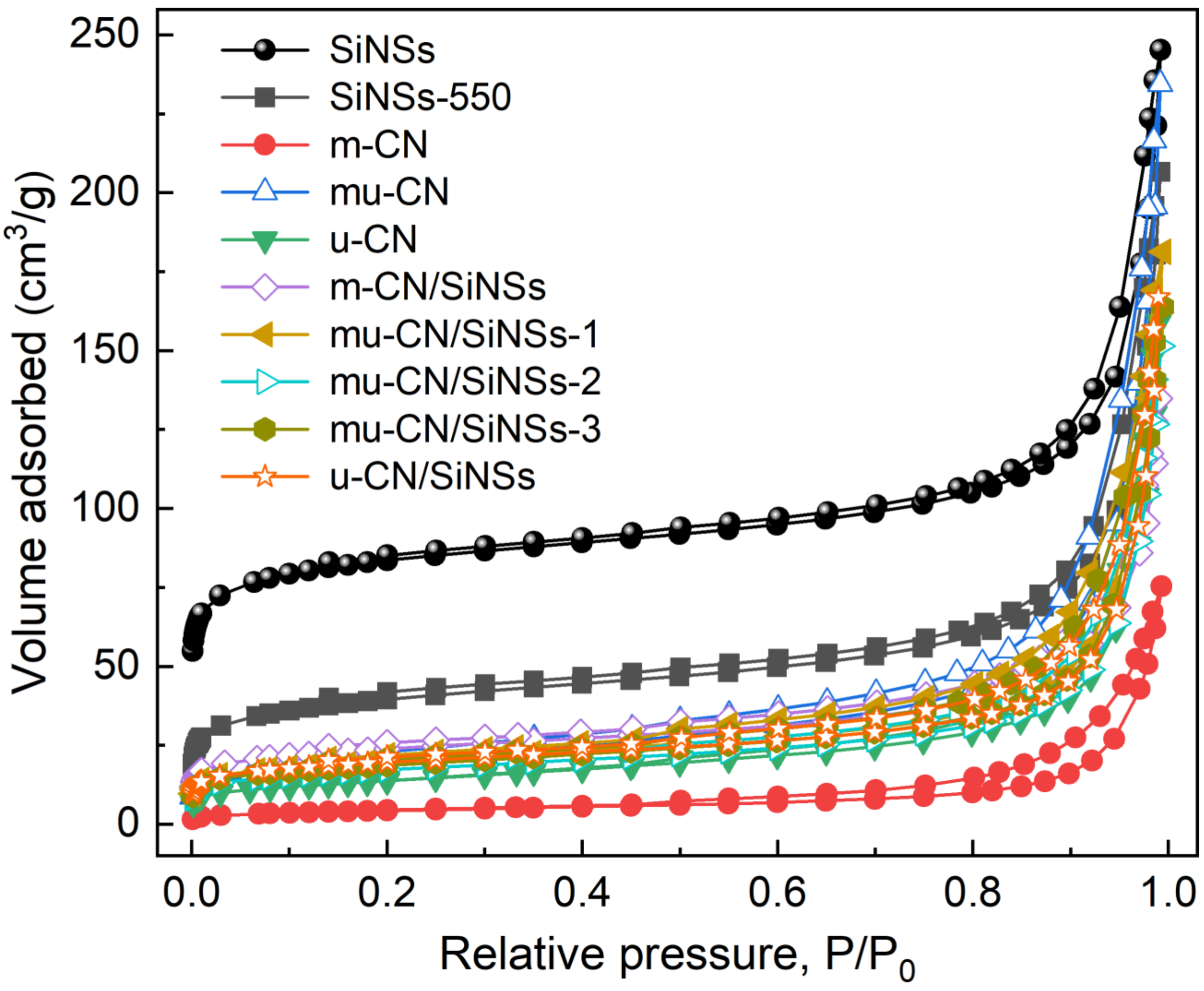

Figure S1. N_2 adsorption-desorption isotherms of the samples.

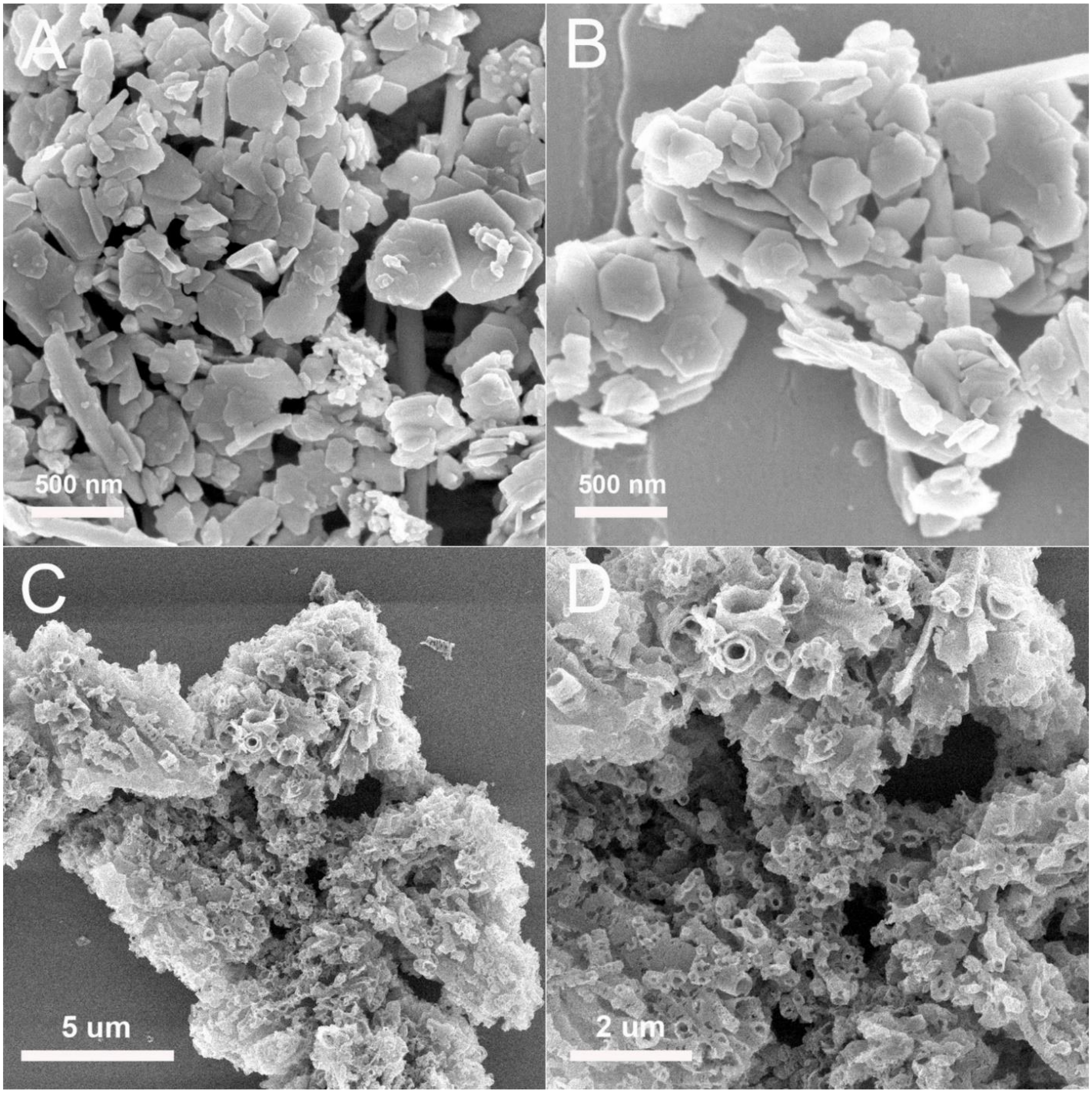

Figure S2. SEM images of (A) SiNSs, (B) SiNSs-550, and (C, D) um-CN.

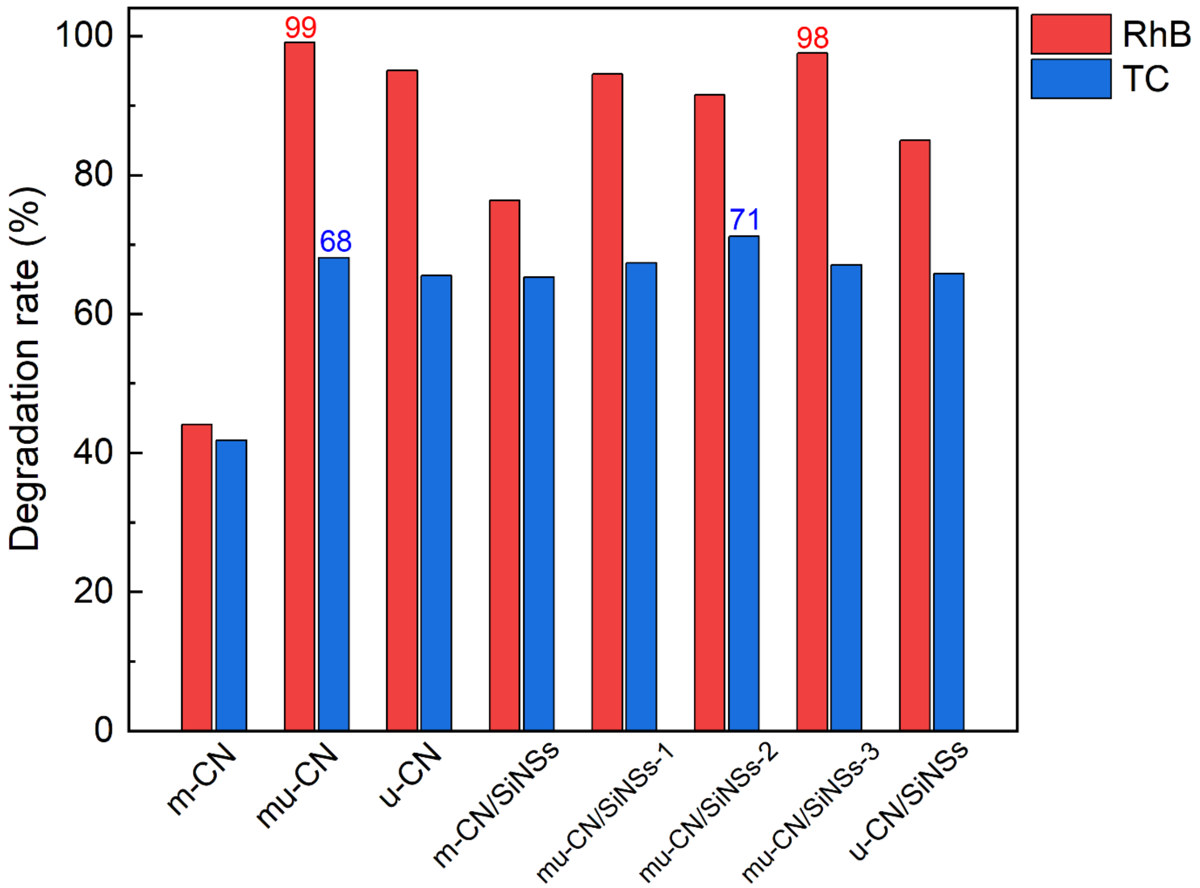

Figure S3. Degradation rates of RhB within 20 min and TC within 50 min over the photocatalysts.

References

1. Sun, Z.; Yao, G.; Zhang, X.; Zheng, S.; Frost, R.L. Enhanced visible-light photocatalytic activity of kaolinite/g-C_3N_4 composite synthesized via mechanochemical treatment. *Appl. Clay Sci.* **2016**, *129*, 7-14.
2. Hao, Q.; Niu, X.; Nie, C.; Hao, S.; Zou, W.; Ge, J.; Chen, D.; Yao, W. A highly efficient g-C_3N_4/SiO_2 heterojunction: the role of SiO_2 in the enhancement of visible light photocatalytic activity. *Phys. Chem. Chem. Phys.* **2016**, *18*, 31410-31418.
3. Jiang, L.; Yuan, X.; Zeng, G.; Wu, Z.; Liang, J.; Chen, X.; Leng, L.; Wang, H.; Wang, H. Metal-free efficient photocatalyst for stable visible-light photocatalytic degradation of refractory pollutant. *Appl. Catal. B* **2018**, *221*, 715-725.
4. Liu, J.; Song, Y.; Xu, H.; Zhu, X.; Lian, J.; Xu, Y.; Zhao, Y.; Huang, L.; Ji, H.; Li, H. Non-metal photocatalyst nitrogen-doped carbon nanotubes modified mpg-C_3N_4: facile synthesis and the enhanced visible-light photocatalytic activity. *J. Colloid Interface Sci.* **2017**, *494*, 38-46.
5. Shi, L.; Liang, L.; Ma, J.; Wang, F.; Sun, J. Remarkably enhanced photocatalytic activity of ordered mesoporous carbon/g-C_3N_4 composite photocatalysts under visible light. *Dalton Trans.* **2014**, *43*, 7236-7244.
6. Yan, Z.; Yang, M.; Chen, Y.; Li, T.; Jing, Q.; Liu, P. Hydroxyl-rich porous silica nanosheets decorated with oxygen-doped carbon nitride nanoparticles for photocatalytic degradation of Rhodamine B. *ACS Appl. Nano Mater.* **2022**, *5*, 818-831.
7. Jun, Y.-S.; Lee, E.Z.; Wang, X.; Hong, W.H.; Stucky, G.D.; Thomas, A. From Melamine-Cyanuric Acid Supramolecular Aggregates to Carbon Nitride Hollow Spheres. *Adv. Funct. Mater.* **2013**, *23*, 3661-3667.
